# A Causal and Inverse Relationship between Plant-Based Diet Intake and in a Two-Sample Mendelian Randomization Study

**DOI:** 10.3390/foods12030545

**Published:** 2023-01-26

**Authors:** Sunmin Park

**Affiliations:** Department of Food and Nutrition, Obesity/Diabetes Research Center, Hoseo University, Asan 31499, Republic of Korea; smpark@hoseo.edu

**Keywords:** plant-based diet, Mendelian randomization, metabolic syndrome, abdominal obesity, causal relationship

## Abstract

A plant-based diet (PBD) has been reported to be linked to metabolic syndrome (MetS) risk in observational studies, but not in causal association studies. We aimed to examine the hypothesis that high PBD exhibited a causal and inverse association with MetS and its components using two-sample Mendelian randomization (MR). PBD was clustered according to food intake, which was assessed by semi-quantitative food frequency questionnaires using a principal component analysis. The instrumental variables were generated using the genome-wide association study (GWAS) of a High-PBD group (≥67th percentiles) after adjusting for the covariates related to MetS, with a significance level of *p* < 5 × 10^−5^ and linkage disequilibrium (r^2^ < 0.001), in a city hospital-based cohort (*n* = 58,701). The causal association of the PBD intake with MetS risk was examined with a two-sample MR approach in the rural plus Ansan/Ansung cohorts (*n* = 13,598). The High-PBD group showed higher energy, fat, protein, cholesterol, fiber, sodium, calcium, vitamin C and D, and flavonoid intake than the Low-PBD group. The High-PBD group showed a lower risk of MetS, waist circumference, hyperglycemia, hypo-HDL-cholesterolemia, and hypertriglyceridemia using an inverse-variance weighted method (*p* < 0.05). Low-PBD intake significantly elevated only waist circumference in weighted-median analysis (*p* < 0.05). No heterogeneity, horizontal pleiotropy, or single genetic variant influenced the causal relationship. In conclusion, low PBD appeared to be causally positively related to MetS risk and its components, but not hypertension. Therefore, Korean-style PBD may be beneficial for decreasing MetS risk in Asian adults.

## 1. Introduction

Metabolic syndrome (MetS) is defined as a congregation of abdominal obesity, hypertension, hypertriglyceridemia, hypo-HDL-cholesterolemia, and hyperglycemia, according to the 2005 revised National Cholesterol Education Program—Adult Treatment Panel III criteria for Asians [1]. MetS contributes to raising coronary heart disease (CHD), type 2 diabetes (T2DM), and stroke. MetS is characterized by insulin resistance. The global prevalence of MetS ranges from 20 to 25% in the adult population [2]. Although traditionally, Asians have been less obese than Caucasians, obesity has been markedly rising among the Asian population in recent decades [3]. Obesity risk depends on two crucial mutually-interacting factors: genetics and lifestyle [4,5]. The conventional diet of our Asian ancestors primarily included unprocessed grains and vegetables, and this, combined with an active lifestyle, contributed to their high insulin sensitivity. However, a shift to a westernized way of living, with increased consumption of processed grains, meat, and fewer vegetables, as well as a more sedentary lifestyle, modulate insulin release patterns, resulting in a dramatic rise in insulin resistance and, consequently, MetS [6,7].

Observational studies have found that lifestyle-related risk factors are associated with MetS risk in various ethnic populations [8]. Healthy lifestyle scores show a lower odds ratio (OR) for MetS risk [1,8], and the primary predictors are alcohol consumption, fruit and vegetable consumption, smoking, and physical activity. Over a 2-year study period in the Heart of New Ulm (HONU) Project, 12% of adults developed MetS, and its incidence was associated with lower optimal lifestyle scores after adjusting for age, gender, education, baseline lifestyle score, cardiovascular disease, and diabetes. This retrospective cohort study suggested that a decrease in optimal lifestyle scores over two years was associated with significantly higher ORs, increased by 2.92 times, of incident MetS compared to a stable lifestyle score. [9]. Several studies have also demonstrated the association of specific nutrient intake with MetS risk [10,11]; however, it is difficult to integrate each nutrient into the regular diet in real life in a sustained manner for long periods. The Dietary Approaches to Stop Hypertension (DASH) and Mediterranean diets are known to lower MetS risk [12,13]. Both diets are rich in whole grains, fish, vegetables, fruits, low-fat dairy products, nuts, and vegetable oils, particularly olive oil, and are limited in red meats, refined and processed foods, and sugar-sweetened foods and beverages.

When the Korean diet is clustered into four dietary patterns, such as a plant-based diet (PBD), a Korean style-balanced diet (KBD), a rice-main diet (RMD), and a Western-style diet (WSD), PBD includes more beans, potatoes, sweet potatoes, eggs, milk, fruits, and nuts than other groups. KBD contains more vegetables, meat, fish, seafood, seaweed, kimchi, and pickles. WSD includes noodles, bread, fast foods, red meats, and soup, while RMD mainly contains rice. PBD is close to the DASH diet in Koreans. In the Korean Genome and Epidemiology Study (KoGES), although high intakes of KBD and PBD mitigated the risk of MetS and its components, only PBD was inversely associated with MetS risk [14,15]. However, the previous studies did not demonstrate the causal relationship between PBD and the alleviation of MetS risk.

It was, therefore, worthwhile to examine the hypothesis that PBD had a causal inverse relationship with the risk of MetS and its traits. In the present study, we evaluated the hypothesis by a two-sample Mendelian randomization analysis in a large Korean cohort from the KoGES. The current study also examined the differences in nutrient intake between the PBD and KBD and determined the nutrients that could raise MetS risk. The results can be applied to prevent MetS by modifying Asian dietary patterns.

## 2. Methods

### 2.1. Participants

A total of 72,299 adults aged over 40, who volunteered to be part of the Korean Genome and Epidemiology Study (KoGES) organized by the Korea Centers for Disease Control and Prevention (KCDC) from 2004 to 2013, were included in the present study. Among the KoGES, the volunteers belonged to the urban hospital-based cohorts (*n* = 58,701), Ansan/Ansung (*n* = 5493), and rural (*n* = 8105) [16]. Based on compliance with the Declaration of Helsinki, the KoGES was approved by the Institutional Review Board (IRB) in KCDC (KBP-2019-055) and IRB in Hoseo University (1041231-150811-HR-034-01) [16]. The volunteers signed informed consent forms [16].

### 2.2. Measurement of Clinical Parameters

The participants were interviewed to elicit information about age, gender, education, income, alcohol intake, smoking status, and physical activity [17]. Family income was divided into three categories: < USD 2000, USD 2000–$4000, and > USD 4000 per month [18]. Education was stratified into < high school, high school, and ≥college [18]. Current smokers were defined based on smoking over 100 cigarettes during their lifetime, but smokers who had not smoked for the last six months were considered past smokers [18]. Daily alcohol intake (g) was calculated by multiplying the alcohol contents, amount, and frequency of alcohol drinking was categorized into 0, 0.1–19, and ≥20 g/day [19].

The anthropometric measurements and blood of the participants were collected for biochemical assay after fasting for more than 12 h. The participants’ height, body weight, and waist circumference were measured, and body mass index (BMI) was calculated as previously described [20]. We calculated the appendicular skeletal muscle mass and fat mass in the city hospital-based cohort from a predicting model generated by a machine learning approach, using dual-energy X-ray absorptiometry (DEXA) in the Ansan/Ansung cohort. As described previously, the skeletal muscle index (SMI) was calculated by dividing appendicular skeletal muscle mass by height [17]. Glucose and lipid profiles in the serum or plasma were measured using an automatic analyzer (Hitachi 7600, Hitachi, Tokyo, Japan) [17]. The hemoglobin A1c (HbA1c) in the EDTA-treated blood was assessed, and serum high-sensitive C-reactive protein (hs-CRP) levels were measured with an ELISA kit (R&D system, Minneapolis, MN, USA) as described previously [17]. The diastolic blood pressure (DBP) and systolic blood pressure (SBP) were assessed three times in the right arm at the heart level of the participant in a sitting position. The average value was used for further analysis.

### 2.3. Food and Nutrient Intake and Dietary Patterns

A semi-quantitative food frequency questionnaire (SQFFQ) containing 103 foods was generated to measure the usual Korean food intake, and then validated [21]. The usual food consumption of the participants was determined using the SQFFQ over six months, and the nutrient intake was calculated from the SQFFQ results using the computer-aided nutrition analysis program (CAN Pro) 3.0 [21].

Foods in SQFFQ were classified into 29 food groups, and the number of dietary patterns was assigned to four using principal component analysis (PCA) based on an eigenvector greater than 1.5 [17]. Four dietary patterns were assigned to meet the criteria in the PCA analysis [17]. The primary foods in each pattern were selected using the orthogonal rotation procedure and food groups with ≥0.40 factor-loading values [14]. Table 1 lists the factor-loading values of the predefined 29 food groups in each dietary pattern. The food groups with high factor-loading values indicated the predominant foods consumed by the participants according to the diet pattern. Four dietary patterns were named PBD, KBD, RMD, and WSD, according to the food groups with higher factor-loading values.

The dietary inflammatory index (DII) was calculated by multiplying the inflammatory weights of foods and nutrients by their intake. As described previously, the equation included four food products, energy intake, thirty-two nutrients, four spices, and caffeine [17].

### 2.4. DNA Genotyping and Quality Control

The Genetic Science Center at the Korea National Institute of Health (NIH, Osong, Korea) conducted genotyping of the participants in the KoGES. In brief, genotyping was conducted using the genomic DNA extracted from the whole blood using a Korean chip (K-chip), which was purchased from Affymetrix (Santa Clara, CA, USA). The K-Chip was generated with 830,000 single nucleotide polymorphisms (SNPs) related to the genetic risk of metabolic diseases (diabetes, hypertension, dyslipidemia, osteoporosis, kidney disease, and autoimmune diseases) and cancer in Koreans [22]. The accuracy of the genetic variants was assessed with the Mahalanobis distance (BRLMM) genotyping algorithm [23]. The genotyping data were obtained from the Korea Biobank (Osong, Korea) after being approved by the Hoseo University Institutional Review Board. The quality of genetic variants was checked for the satisfaction of the criteria, including (1) genotyping accuracy (≥98%), (2) low heterozygosity (<30%), (3) missing genotype call rates (<4%), (4) minor alleles frequency (MAF; >1%), (5) Hardy–Weinberg Equilibrium (HWE; *p* > 0.05), and (6) no gender bias [24]. Furthermore, the imputed genetic variants were selected to meet the criteria of a posterior probability score ≥0.90 and high informative content of the genotype (≥0.5).

### 2.5. Experimental Design for a Two-Sample MR Design

**Figure 1:**
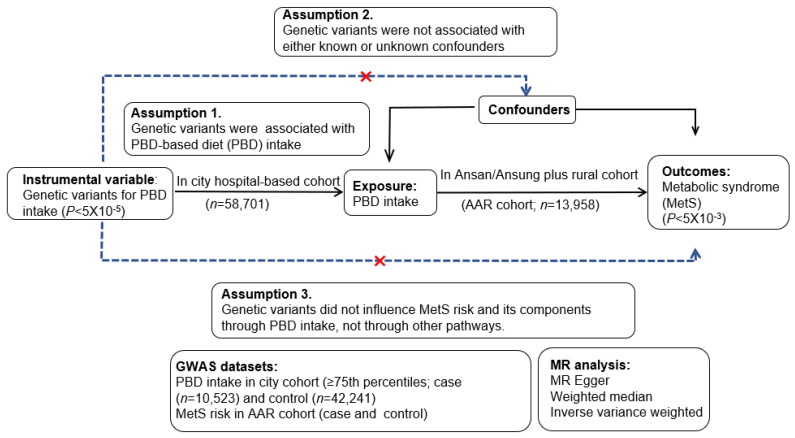
The scheme and assumptions to examine the causal association of plant-based diet (PBD) intake with metabolic syndrome (MetS) and its traits using two-sample Mendelian randomization (MR) analysis. The present study met all three assumptions of the MR analysis.

The causal association of the PBD intake with the risk of MetS and its traits was estimated with a two-sample MR. The genetic variants predominantly involved in the PBD intake were searched from a genome-wide association study (GWAS). An instrumental variable was selected based on a significance level of 5 × 10^−5^ and no association with confounding factors (Figure 1). The two-sample MR analysis was designed to confirm the relationship based on the assumptions (Figure 1): (1) instrumental variables were the genetic variants associated with the PBD intake; (2) they were not associated with confounding factors; (3) they did not affect the risk of MetS nor its components [16].

A two-sample MR included weighted median, inverse variance weighted (IVW), and MR-Egger estimator approaches. The IVW method was considered the primary causal effect estimate. It is a practical analysis technique, assuming that all genetic variants are effective instrumental variables and have a robust ability to detect causality [17,19]. On the other hand, the IVW approach requires exposure to genetic variants to influence the target outcome alone. The genetic variants associated with confounding factors were eliminated from the instrumental variable to satisfy the criteria of two-way MR analysis [17,19]. The causal association of the PBD intake with the risk of MetS and its components was considered stable and reliable if the three approaches in the MR analysis generated equivalent estimates of the causality.

### 2.6. Instrumental Variables in a Two-Sample MR Analysis

The GWAS was performed with the low and high PBD intakes using the PLINK version 2.0, downloaded from http://pngu.mgh.harvard.edu/~purcell/plink (accessed on 23 November 2021), in the urban hospital-based cohort (*n* = 58,701). The cutoff was divided into tertiles in the MR analysis, since the minimum intake of 67th percentiles showed a beneficial impact on MetS risk in the present and previous studies [19,25]. The PBD intake was classified into low and high intake groups, with the 67th percentile as the point of division [19,25]. The participants having ≥67th percentile of PBD intake were considered the case (High-PBD; *n* = 11,740), and the rest of them were the control (Low-PBD; *n* = 46,961). The association of genetic variants with PBD intake was determined using logistic regression adjusted with covariates. Age, gender, residence area, BMI, energy intake, exercise, smoking, and the education of the participants were incorporated into covariates for GWAS. The logarithm of statistical significance levels for the genetic variants associated with PBD was plotted according to the chromosome orders (Manhattan plot). The quantile–quantile (Q-Q) plot exhibited the deviation of the logarithm of observed *p* values of SNPs from their expected ones, which were calculated using a theoretical χ^2^ distribution, as described previously [17]. The gene names of the selected SNPs were identified by seeking the g: profiler database (https://biit.cs.ut.ee/gprofiler/snpense, accessed on 21 December 2021) [17]. The selected genetic variants were used as instrumental variables, which are present in Supplementary Table S1.

The PBD-related genetic variants showed no *p*–value of 5 × 10^−8^, which may be related to a lower association of dietary intake with genetics. The statistical significance level of < 5 × 10^−5^ was applied, which has been used to explore genetic variants related to lifestyles in GWAS [26,27]. The liberal statistical threshold can be allowed since many SNPs depend on linkage disequilibrium (LD) relation [26,27]. The LD of all selected SNPs within a 10 MB distance was checked at r^2^ < 0.001, similar to D’ < 0.2, using the R program, and those with LD relations were eliminated. The instrumental variables should not be associated with outcomes such as MetS and its components to satisfy assumption 3 in a two-sample MR design (Figure 1). Therefore, the instrumental variables are appropriate for a two-sample MR study.

In the rural plus Ansan/Ansung cohort (*n* = 13,598), the association of the instrumental variables with the MetS risk, including its components, was evaluated (Figure 1). If they were significantly linked to the MetS risk at *p* < 5 × 10^−3^, they were excluded from satisfying the criteria of the two-sample MR analysis. 

### 2.7. Statistical Analysis

Statistical analysis was conducted using SAS (Cary, NA, USA). Descriptive statistics for the categorical variables were obtained using the frequency distribution between the Low and High-PBD intake groups [28,29]. Their significant differences were analyzed using the chi-square test. Descriptive statistics of the continuous variables were determined with mean and standard deviation, according to the Low- and High-PBD groups [28,29]. The differences in the continuous variables were analyzed by analysis of covariance (ANCOVA) to adjust for the covariates. Multiple comparisons were performed with Tukey’s test.

Two-sample MR (version 0.4.26) and MR packages (version 0.5.1) in the R program were applied for conducting the two-sample MR analysis, which determined the causal relationship of the PBD intake (exposure) to MetS and its components (outcomes). In the IVW method, the Wald ratios for each genetic variant (instrumental variables) were calculated. The X and Y axes indicated the PBD intake as the exposure and MetS and its components as the outcome, respectively, in the Ansan/Ansung plus rural cohort.

## 3. Results

### 3.1. Characteristics of the Urban Hospital-Based Cohort According to PBD Intake and Gender

Women comprised a higher proportion of the High-PBD group than men, and gender influenced the MetS somewhat differently (Table 2). The participant characteristics were classified by gender and PBD intake. The participants in the High-PBD group were older, comprised more women, earned higher incomes, and were more educated than those in the Low-PBD group. They also smoked less, consumed less alcohol, and exercised more regularly, suggesting that they lived healthy lives (Table 2). The participants on the High-PBD had a higher energy intake, which was calculated as the ratio of estimated energy requirement (EER); lower carbohydrate intake; and higher protein, fat, and cholesterol intake than those on the Low-PBD (Table 2). The fatty acid intake, including saturated, monounsaturated, and polyunsaturated fatty acids, also showed an identical pattern to total fat intake. Calcium, sodium, and fiber intakes were higher in the High-PBD group than in the Low-PBD group. However, sodium intake between the two groups was similar when calculated based on daily energy intake (Table 2). As expected, vitamin C, vitamin D, and flavonoid intake significantly differed between the High-PBD and the Low-PBD groups (Table 2). The DII showed an inverse trend to the antioxidant nutrient intake.

### 3.2. Metabolic Parameters Related to MetS

MetS incidence was lower in the High-PBD group than in the Low-PBD group, and the PBD intake was inversely associated with MetS risk after adjusting for covariates (Table 3). BMI and SMI were higher in the Low-PBD group than in the High-PBD group only in women, while waist circumference and fat mass were lower in the High-PBD group for both genders (Table 3). This suggests that High-PBD decreases both skeletal muscle and body fat mass in women, but only body fat mass in men. The PBD intake was inversely linked to BMI, waist circumference, SMI, and fat mass (Table 3). Therefore, women’s BMI decrement in the High-PBD group was related to the decline in skeletal muscle mass. However, men’s BMI and skeletal muscle mass showed no significant difference between the Low-PBD and High-PBD groups.

The participants in the Low-PBD group exhibited higher serum glucose and HbA1c levels than those in the High-PBD group (Table 3). The PBD intake was significantly and inversely associated with serum glucose, HbA1c concentrations, and insulin resistance (Table 3). The PBD intake showed a positive association with serum HDL concentrations and an inverse association with serum triglyceride concentrations (Table 3). DBP, but not SBP, showed a similar trend in serum glucose concentrations. The PBD intake was positively associated with the estimated glomerular filtration rate (eGFR). However, the PBD intake was not associated with serum alanine aminotransferase (ALT), aspartate transaminase (AST), or total bilirubin concentrations (Table 3).

### 3.3. Association of PBD with MetS and its Components in Observational Studies

**Figure 2:**
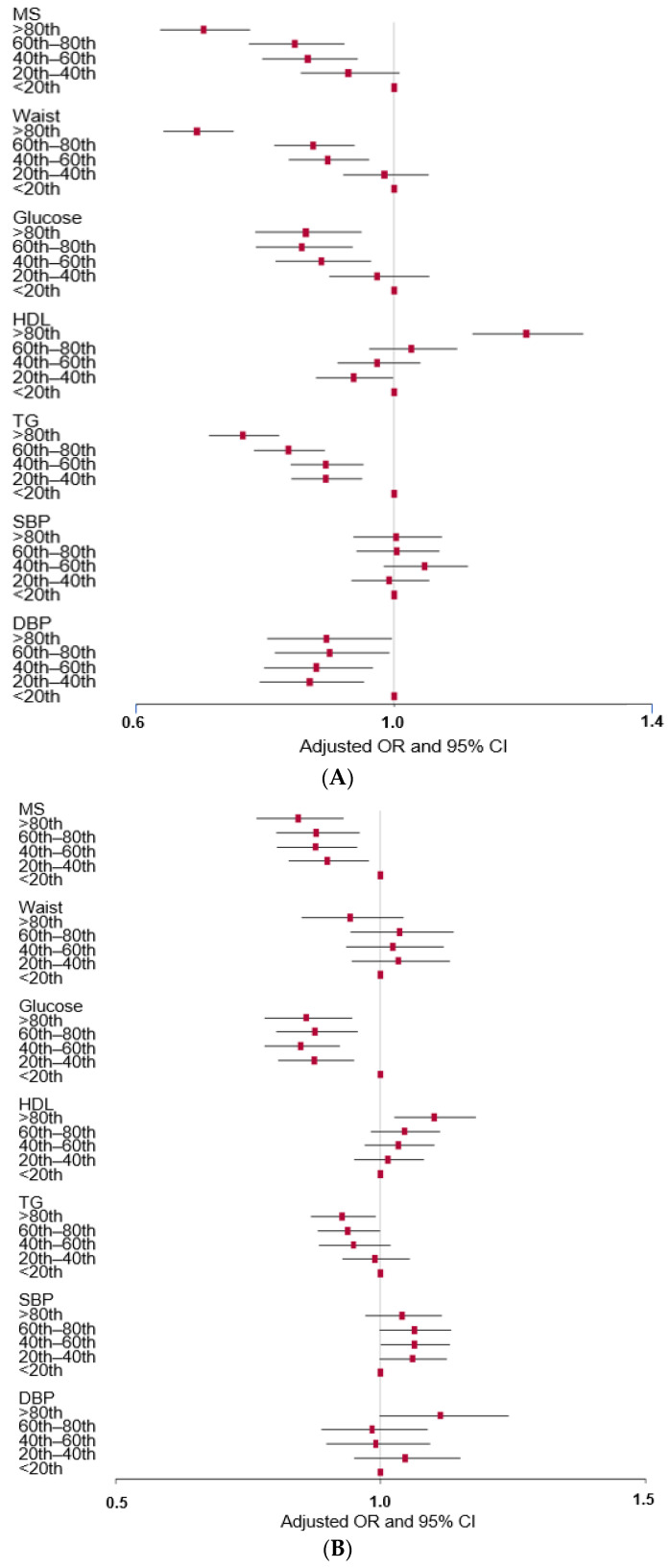

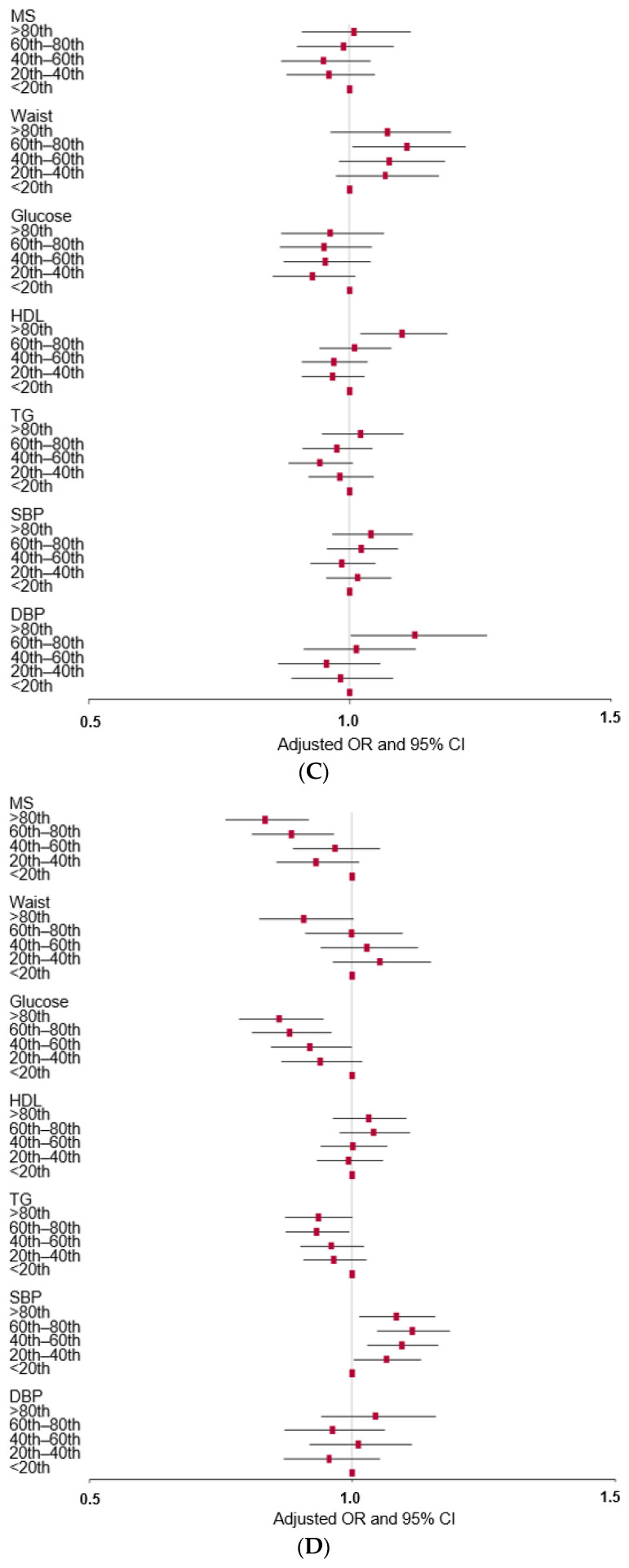
Association of plant-based diet (PBD) intake with metabolic syndrome (MetS) risk and its traits according to observational estimates in an urban hospital-based cohort. (**A**) Association of the quantiles of PBD intake with metabolic syndrome and its components; (**B**) association of the quantiles of vitamin C intake with metabolic syndrome and its components; (**C**) association of the quantiles of dietary fiber with metabolic syndrome and its components; (**D**) association of the quantiles of flavonoid intake with metabolic syndrome and its components. OR, odds ratio; CI, confidence intervals. The reference was the Low-PBD intake (<67th percentile).

The adjusted OR of MetS risk was significantly higher in the low percentiles of PBD intake than in the High-PBD (≥80th percentile), and the participants with Low-PBD (<20th percentile) were at a risk of MetS 1.421 times higher compared to the High-PBD group. The waist circumference, serum glucose concentrations, triglyceride concentrations, and DBP showed inverse associations with PBD intake, whereas the serum HDL concentrations were positively related to it (Figure 2A). Figure 1 presents the association of predominant components in PBD, such as vitamin C, flavonoids, and dietary fiber, with MetS risk. Vitamin C intake was inversely associated with MetS risk and serum triglyceride and glucose concentrations, while it was positively associated with serum HDL concentrations (Figure 2B). The association between the flavonoid intake and the risk of MetS and its components exhibited similar trends as the vitamin C intake (Figure 2C). However, the fiber intake did not have any such associations with MetS and its components (Figure 2D). From these results, a level in approximately the 60th–80th percentile range can be determined as the threshold for the association between PBD intake and MetS risk. Therefore, the 67th percentile for tertiles was used for the cut-off point to categorize PBD intake.

### 3.4. Instrumental Variables Generation for a Two-Sample MR Analysis

**Figure 3:**
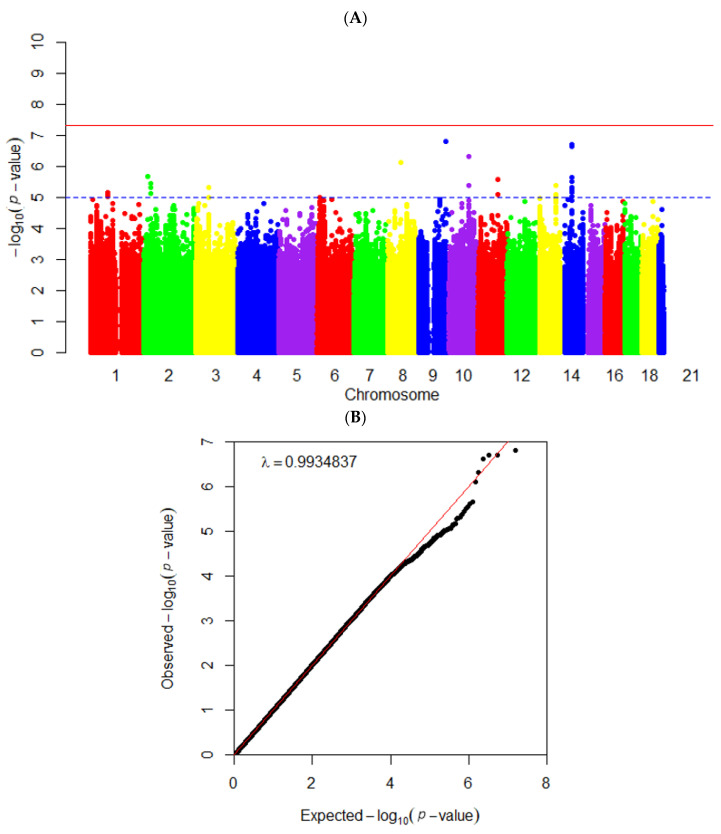
Manhattan and quantile–quantile (QQ) plots for single nucleotide polymorphism (SNP) association with a plant-based diet intake. (**A**) Manhattan plot; (**B**) QQ plot. The PBD intake was categorized into Low- and High-PBD groups, differentiated at the 67th percentiles. Genetic variants of the PBD intake were determined with a genome-wide association study (GWAS).

The case was assigned to ≥67 percentiles of PBD intake according to the results of the observational analysis and previous studies. The Manhattan plot of the GWAS results with PBD intake showed the log of *p* values of the genetic variants in the order of chromosomes (Figure 3A). Significant SNPs were clustered on chromosomes 2, 9, 10, 11, and 14 (Figure 3A). In Figure 3B, most of the observed and expected *p* values in the Q-Q plot were placed on the midline, indicating that the observed and expected values were similar. The genome inflation factor (λ) was 0.994 (Figure 3B), suggesting no severe bias of the genetic variants from the GWAS results.

The instrument variables were selected from the genetic variants resulting from the GWAS of the PBD intake with *p* < 5 × 10^−5^. Those with potential LD were deleted based on an r^2^ threshold of 0.001, within a distance of 10,000 kb, in the LD analysis. Forty-two genetic variants met the criteria of the instrument variables for a causal association of the PBD intake with the risk of MetS and its components. The 42 SNPs’ characteristics are listed in Supplemental Table S1. The 42 genetic variants were used as the instrument variables in a two-sample MR analysis.

### 3.5. Causal Association of PBD Intake, MetS by a Two-Sample MR Analysis

**Figure 4:**
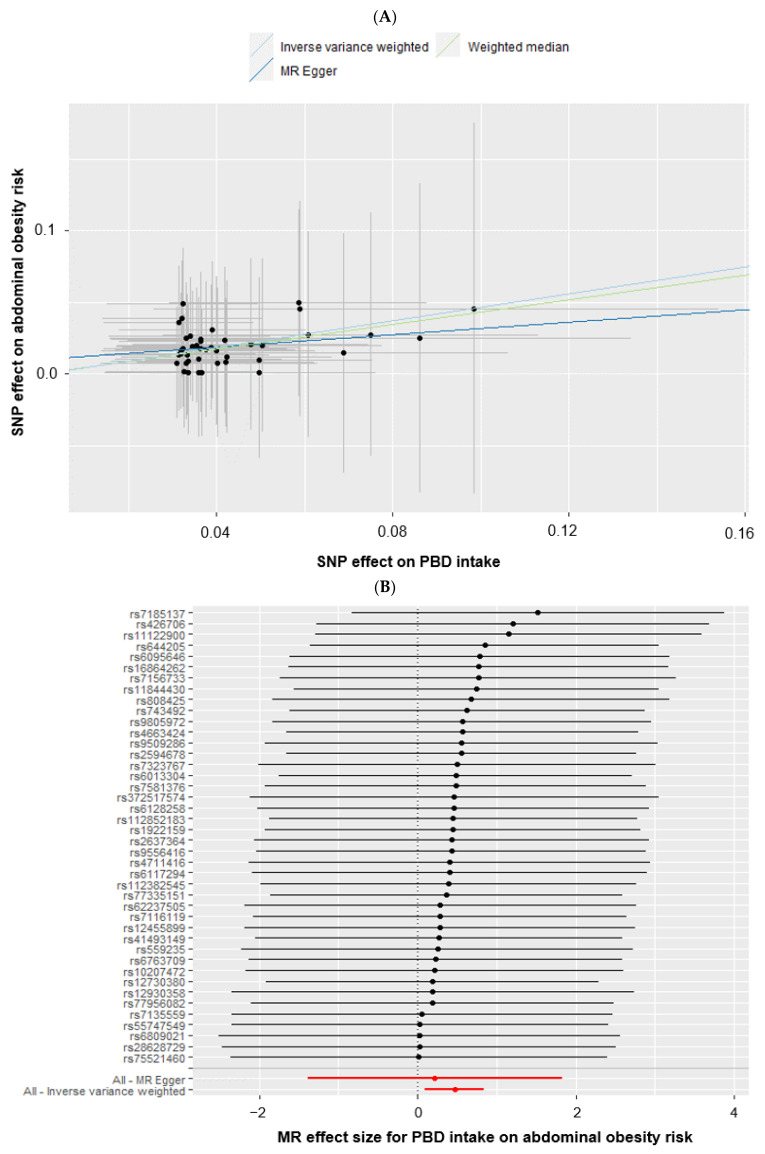

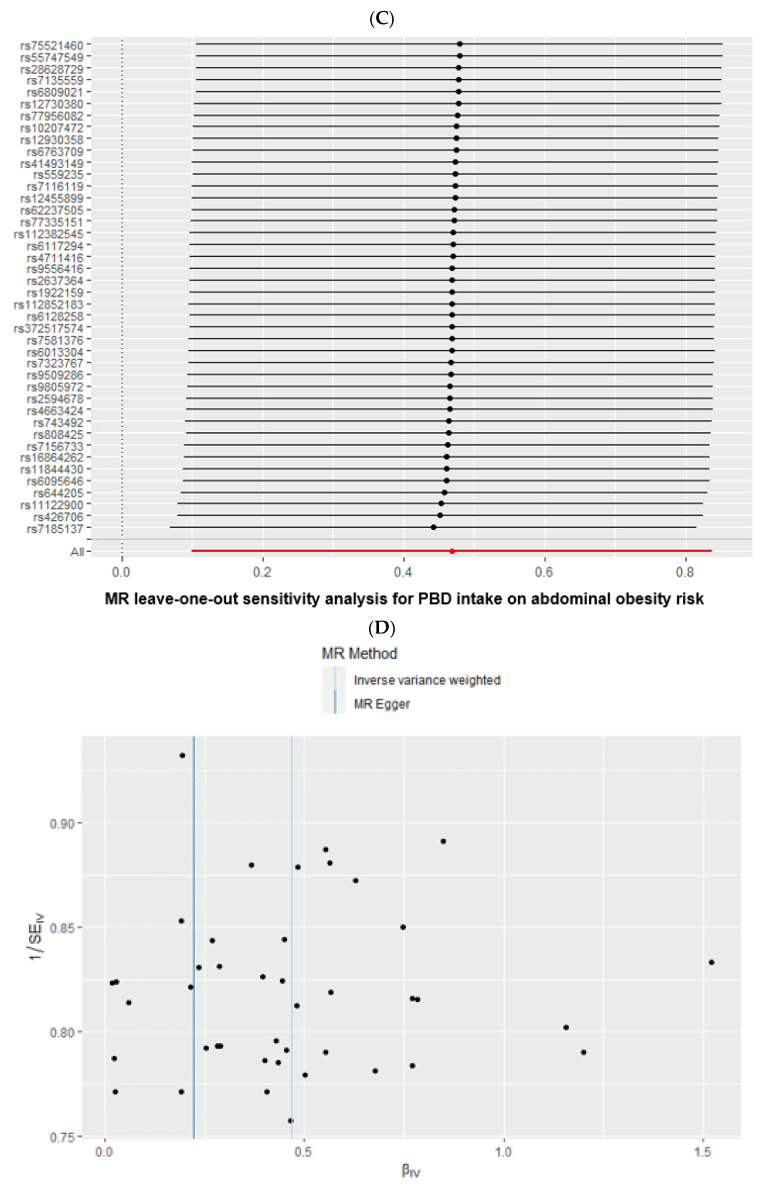
Association between plant-based diet (PBD) intake (cutoff: 67th percentiles) on abdominal obesity (AB) risk, determined by waist circumference (men, ≥90 cm and women, ≥85 cm) using a two-sample Mendelian randomization (MR) analysis. (**A**) Correlation between the effect size of the single nucleotide polymorphism (SNP)-PBD intake (x-axis, standard deviation (SD) units) and SNP-AB association (y-axis, log OR) to the SD bars. (**B**) Forest plot between PBD intake and AB. Each black dot represents an increase in the log OR of each SD in AB, using each SNP of PBD intake as a separate instrumental tool in the MR-Egger method. Both MR–Egger and inverse-variance weighting (IVW) indicated a combined causal estimate with all SNPs in a single instrumental variable, using random effects of MR-Egger and IVW. The horizontal lines in each SNP represented 95% confidence intervals. (**C**) Leave-one-out sensitivity analysis of MR for the dietary PBD intake on the AB. Each black dot indicates an IVW method for estimating the causal effect of PBD intake on AB, from which the single SNP was excluded. The IVW estimate was described using eight SNPs in the leave-one-out sensitivity analysis. (**D**) IVW and MR-Egger regression funnel plots for the PBD intake on the AB. The vertical line represented a causal estimate, using all SNPs combined into a single instrumental variable, for each IVW and MR-Egger method.

The causal association of PBD intake with MetS using four different MR estimator methods in a two-sample MR analysis is summarized in Table 3. In the IVW analysis, the Low-PBD intake (<67th percentile) was causally associated with the MetS risk (OR = 1.422, 95% CI = 1.047–1.930; *p* = 0.024), waist circumference (β = 0.469, 95% CI = 0.100–0.837; *p* = 0.013), hyperglycemia (β = 0.542; 95% CI = 0.099–0.985; *p* = 0.017), hyper-triglyceridemic (β = 0.427; 95% CI = 0.085–0.768; *p* = 0.014), and hypo-HDL-cholesterolemia (β = 0.343; 95% CI = 0.061–0.626; *p* = 0.017) (Table 4). The Low-PBD intake was causally and positively associated with the waist circumference only in the weighted median method (Table 4). As seen in Figure 4A, the intercept of the association of the SNP effects of abdominal obesity, represented by waist circumference and Low-PBD intake, passed zero in the IVW and weighted median methods, indicating that genetically predicted waist circumference was causally associated with PBD intake. The association between the PBD intake and abdominal obesity determined with waist circumference was shown using each instrumental variable in the MR-Egger analysis and all the instrumental variables in the MR-Egger and IVW methods (Figure 4B). The Low-PBD intake exhibited a causative and positive relationship with abdominal obesity among the MetS components using the IVW method. However, there was no significant association using the MR-Egger analysis (Figure 4B).

Cochran’s Q statistics represent the heterogeneity of the association between PBD intake, MetS, and its components, indicating no evidence of heterogeneity in the association (Table 4). The intercept of the horizontal pleiotropy was close to zero in the MR-Egger intercepts (*p* = 0.778; Table 4), suggesting no horizontal pleiotropy between PBD intake and MetS risk (Table 4). There was also no horizontal pleiotropy in the MetS components, including waist circumferences, serum triglyceride, HDL, glucose concentrations, and blood pressure. In the leave-one-out method, the deletion of a single SNP from the instrumental variables did not alter the causal relationship between the PBD intake, MetS, and its traits (Figure 4C). The plot of the MR estimate of each SNP with waist circumference is present in Figure 4C, indicating a causal association of the MAF-corrected genetic variant with waist circumference. Because the MAF was likely to be related to precision, the MAF correction was proportionally applied in proportion to the standard error of the genetic variants for MetS. The funnel plot of MR-Egger regression for waist circumference showed symmetry in the IVW method, but asymmetry in the MR–Egger method (Figure 4D). Therefore, the PBD intake exhibited a causal and inverse association with MetS and its components, including waist circumference and serum glucose, HDL, and triglyceride concentrations, using the IVW method.

MR, Mendelian randomization; PBD, plant-based diet; AB, abdominal obesity; SNP, single nucleotide polymorphism; IVW, inverse-variance weighting.

## 4. Discussion

PBD, similar to the Mediterranean diet, was shown to mitigate MetS and its components in the current study. However, the primary components of the PBD are somewhat different in different countries. The PBD in Koreans predominantly contains beans, potatoes, eggs, green vegetables, seaweed, milk, fruits, and nuts, and it was causally inversely associated with waist circumference. The Korean-style PBD is similar to the Mediterranean diet, but does not include whole grains, fish, and alcohol. The adults on a High-PBD have a higher energy intake than those with Low-PBD based on EER, but their BMI, waist circumference, and fat mass were lower than those on a Low-PBD. However, the PBD intake was inversely related to the SMI. These results indicated that a High-PBD intake appeared to lower BMI, with decreased body fat mass accumulation. Furthermore, the High-PBD decreased the risk of MetS and its traits in the IVW method of two-way MR analysis. Therefore, a High-PBD intake can be causally and inversely related to MetS and its traits, especially waist circumference. This study was novel in its attempt to show the causality of high PBD intake in Asians despite a higher energy intake.

A two-way MR analysis can estimate the causal relationship between PBD intake and MetS risk in observational studies. The instrumental variables for the PBD intake were selected from the GWAS with a liberal statistical significance level (*p* < 5 × 10^−5^), since no genetic variants for PBD intake satisfied the Bonferroni corrections and a substantial number of them showed LD associations. Therefore, the genetic variants with LD relations should be eliminated from the total number, indicating that their number was much smaller than those of the genetic variants in the original chip. Forty-two instrumental variables were selected for the PBD intake (Supplementary Table S1) which were not associated with MetS and its traits. The genetic variants used as instrumental variables were not associated with outcomes such as MetS, its components, and their confounders in the Ansan/Ansung plus rural cohort [30]. These results suggest that the instrumental variables met the criteria for the two-way MR analysis.

The genes of the instrumental variables were *AGBL4*, *RASGRP3*, *SUMF1*, *MBNL1*, *SLC22A9*, *PCDH15*, *PTPRQ*, *SAMD4A*, *RBFOX1*, *ASIC2*, *MEAK7*, *CASC20*, and others, and the genetic characteristics of their genetic variants are given in Supplementary Materials Table S1. Since the genes have not been studied extensively, there was no evidence of their relationship with PBD-related metabolism. The instrumental variables could not explain the PBD mechanism in MetS and its components. Current research demonstrates that PBD intake has a causal relationship with MetS and its traits according to the IVW method, and was causally related only to abdominal obesity, determined by waist circumference, in the weighted median method.

However, the PBD intake did not show any causal relationship with MetS and its traits when the MR-Egger method was used. In addition to causal relations, MR-Egger analysis examines the presence of horizontal pleiotropy in instrumental variables: the outcome for the association between abdominal obesity and PBD intake differs on average from zero, indicating no horizontal pleiotropy [31]. In the present study, although MR-Egger did not show a significant causal association of PBD intake with MetS, it showed no horizontal pleiotropy in MetS and its traits by PBD intake. Consistent with the present study, previous studies have demonstrated an insignificant causal relationship in MR-Egger analysis. No significant causal association in MR-Egger analysis indicated the potential to have an inflated type 1 error in the causal estimates. This may result in decreased statistical power, potentially due to insufficient instrumental variables [29]. However, the overall results suggested that Korean-style PBD intake revealed a causal inverse relationship between MetS and waist circumference.

Dietary pattern modulation has better efficacy in preventing and alleviating metabolic diseases than single nutrient alteration [32]. In observational studies and randomized clinical trials, shifts to the Mediterranean and DASH diets have been reported to improve dyslipidemia in persons with MetS, T2DM, and cardiovascular diseases [33,34]. Study of the Mediterranean diet has revealed that the genetically related metabolic signature of fatty acid and amino acid metabolism is inversely linked to cardiovascular disease risk using the MR analysis in the US Nurses’ Health Studies I and II and the Health Professionals Follow-up Study [35]. Asians have traditionally consumed whole grains, fish, and fermented beans and vegetables, such as those included in the PBD. However, for several decades, diets have changed, with the inclusion of refined grains, processed foods, and meat. In KoGES, the dietary patterns were categorized into KBD, PBD, WSD, and RMD. When dietary patterns are divided into three categories, the PBD belongs to the KBD pattern. The KBD and PBD improved MetS and primarily dyslipidemia, while the PBD alleviated MetS, reduced waist circumference, and improved dyslipidemia and hyperglycemia [36]. In the present study, the participants in the PBD group consumed more beans, potatoes, green vegetables, eggs, milk, fruits, and nuts than the other groups. The PBD diet was comparable to the Mediterranean and DASH diets. However, PBD had a negative relation with alcohol intake in the present study.

While PBDs such as the Mediterranean and DASH diets have shown benefits in lowering MetS risk, other studies have shown inconsistent results regarding the association between PBD and MetS [36,37]. These inconsistencies can be attributed to the difference in the composition of the PBDs [36]. A healthy PBD contains a greater proportion of whole grains, beans, vegetables, fruits, nuts, coffee, and tea, while an unhealthy PBD contains refined grains, potatoes, salty and fried plant foods, sugar-sweetened beverages, and sweets. The unhealthy PBD is positively associated, and the healthy PBD is inversely related, with MetS risk [36]. This indicated that the quality of the PBD is essential to addressing the MetS risk. The present study found that the Korean-style PBD was close to a healthy PBD, rich in potatoes, beans, eggs, milk, green vegetables, fruits, and nuts. The vitamin C, vitamin D, calcium, fiber, and flavonoid intakes were much higher in the High-PBD group than in the Low-PBD group. Beneficial nutrients, as well as sodium intakes, were also higher in the High-PBD group than in the Low-PBD group. A previous two-sample Mendelian randomization (MR) study demonstrated that insufficient vitamin C intake elevates the risk of MetS and its traits, especially hyperglycemia, in Asians [16]. Therefore, the high content of vitamin C, vitamin D, calcium, and flavonoids in the PBD are linked to its action in modulating MetS risk, since they are well-known nutrients that improve the components of MetS [1,16].

Asians, including Koreans, consume rice as a staple food. The RMD, however, is positively associated with MetS risk in this population, which is attributed to a lower intake of other essential nutrients [38,39]. On the other hand, while rice is an essential food in both KBD and PBD, these diets lowered the risk of MetS and its traits due to the lower refined rice content and the addition of various other nutritious foods, which improve insulin sensitivity [14,40]. Furthermore, the participants in the High-PBD group had a higher energy intake from higher fat and protein intakes, but lower carbohydrate intake, than those in the Low-PBD group, although the ratio of carbohydrates, protein, and fat was about 70, 14, and 16 En%, basically constituting a high-carbohydrate diet. Freuer et al. revealed that a diet containing a high proportion of fat and a low proportion of carbohydrates (about 48 En% carbohydrates, 16 En% protein, and 36 En% fat) is causally associated with higher BMI and waist circumference among the Genetic Investigation of Anthropometric Traits Consortium and Europeans in the Social Science Genetic Association Consortium [41]. A relatively high-carbohydrate diet (about 70% carbohydrate and 13–15% fat) is positively associated with MetS, hyperglycemia, and hypertriglyceridemia risk [10,42]. Therefore, MetS risk is associated with the amount and quality of carbohydrates in the diet. Therefore, the quality of carbohydrates in the PBD is crucial in influencing the MetS risk. Additionally, the optimal macronutrient ratio needs to be studied and recommendations need to be made.

The merit of the present study is that it is novel in that it reports that Korean-style PBD intake is causally associated with reduced MetS risk and waist circumference. In contrast, there were some limitations to the present study. First, a liberal statistical significance (*p* < 5 × 10^−5^) was applied when instrumental variables were generated from the GWAS of PBD intake. The reasons were as follows: no genetic variants for the PBD intake satisfied a Bonferroni corrected statistical level (*p* < 5 × 10^−8^), but many SNPs were removed from the analysis due to having high LD. Therefore, a liberal statistical *p*-value has been applied to select the instrumental variables for lifestyle measurements in previous studies [26,27]. The genetic variants are still considered weak instrumental variables due to insufficient statistical evidence related to exposure (PBD intake) [43]. Second, the potential residual pleiotropy remained in the lifestyle-related MR study. However, the MR-Egger intercept was close to zero, indicating that the pleiotropy did not significantly impact the MR results. Finally, the PBD intake was assessed according to the usual food intake estimated from the SQFFQ. Although the SQFFQ for Korean food intake was validated with the 12-day food records [17,44], it could be underestimated or overestimated.

## 5. Conclusions

The Korean-style PBD intake had a positive causal relationship with MetS and its traits, except for hypertension, although daily energy consumption was greater in the High-PBD group than in the Low-PBD group. It suggested that a high PBD intake decreased MetS, abdominal obesity, hyperglycemia, hypertriglyceridemia, and hypo-HDL-cholesterolemia risk. Therefore, we might recommend daily Korean-style PBD to prevent MetS risk in middle-aged adults. Further research will be needed, with large prospective and randomized clinical studies, to validate the effects and mechanism of PBD in lowering MetS risk.

## Figures and Tables

**Table 1:** The factor-loading values of the predefined 29 food groups in each dietary pattern.

Food Groups	Korean Balanced Diet		Plant-Based Diet		Western-Style Diet		Rice-Main Diet	
Rice	−3		−7		4		93	*
Whole grain	9		−4		−2		−93	*
Bread	−7		35		53	*	−5	
Cookie	−7		31		30		6	
Noodles	2		2		62	*	3	
Bean	32		48	*	3		2	
Potato	24		50	*	5		−3	
Kimchi	50	*	1		−1		−3	
Egg	8		43	*	15		5	
Fast food	−4		15		76	*	−4	
Green vegetables	67	*	42	*	−2		−2	
White vegetables	70	*	29		2		2	
Mushroom	49	*	35		−6		−4	
Fatty fish	53	*	23		11		0	
Whitefish	66	*	17		12		0	
Crab	49	*	4		19		1	
Processed meats	18		15		6		−1	
Red meat	46	*	−7		41	*	8	
Chicken	33		−6		39		4	
Soups	16		4		65	*	−5	
Seaweeds	44	*	41	*	−2		−4	
Milk	11		49	*	2		0	
Beverage	20		32		6		2	
Coffee	10		−1		19		15	
Tea	14		−8		24		14	
Fruits	19		48	*	−6		−6	
Pickles	50	*	−1		4		2	
Alcohol	18		−28		16		6	
Nuts	−2		50	*	6		−5	
Variance Explained by Each dietary pattern	3.545		2.487		2.291		1.799	

Values were factor-loading values, and their absolute values were higher than 0.4, indicating the principal component of each diet. The principal components of the food groups are flagged by an ‘*’, and the name of each dietary pattern was assigned from the flagged principal food groups.

**Table 2:** Demographic characteristics and lifestyles according to plant-based diet (PBD) intake status and gender.

	Men		Women	
	Low PBD (*n* = 16,095)	High PBD (*n* = 4198)	Low PBD (*n* = 23,030)	High PBD (*n* = 15,378)
Age (year)	55.6 ± 0.06 ^b^	58.0 ± 0.12 ^a^	51.9 ± 0.05 ^d^	53.1 ± 0.06 ^c***+++###^
Income (Yes, %) ≤USD 2000 USD 2000–4000 >USD 4000	1330 (8.70) 6595 (43.1) 7366 (48.2)	276 (6.86) ^‡‡‡^ 1612 (40.1) 2134 (53.1)	2995 (13.8) 9740 (45.0) 8905 (41.2)	1177 (8.12) ^‡‡‡^ 6245 (43.1) 7065 (48.8)
Education (Yes, %) ≤Middle school High school ≥College	1476 (14.4) 7807 (75.9) 1001 (9.73)	277 (12.6) ^‡‡‡^ 1627 (74.2) 290 (13.2)	4692 (26.1) 13153 (69.1) 931 (4.89)	1776 (15.7) ^‡‡‡^ 8718 (76.8) 851 (7.50)
Smoke (Yes, %)	4779 (29.8)	885 (21.1) ^‡‡‡^	2336 (19.6)	497 (6.57) ^‡‡‡^
Alcohol intake (g/day)	39.6 ± 0.38 ^a^	21.3 ± 0.75 ^b^	6.94 ± 0.33 ^c^	3.09 ± 0.38 ^d***+++###^
Physical activity (Yes, %)	9135 (57.0)	2817 (67.3) ^‡‡‡^	10962 (47.8)	9062 (59.1) ^‡‡‡^
Energy (EER percent)	86.2 ± 0.25 ^d^	106.0 ± 0.47 ^b^	90.0 ± 0.21 ^c^	112.6 ± 0.24 ^a***+++###^
CHO (En%)	72.0 ± 0.06 ^b^	69.3 ± 0.11 ^d^	73.1 ± 0.05 ^a^	70.0 ± 0.06 ^c***+++##^
Protein (En%)	13.0 ± 0.02 ^c^	14.0 ± 0.04 ^b^	13.0 ± 0.02 ^c^	14.2 ± 0.02 ^a**+++##^
Fat (En%)	13.6 ± 0.04 ^b^	16.2 ± 0.08 ^a^	12.5 ± 0.04 ^c^	15.6 ± 0.04 ^a***+++###^
Saturated fat (En%)	4.39 ± 0.02 ^b^	4.94 ± 0.04 ^a^	4.04 ± 0.02 ^c^	4.85 ± 0.02 ^a**+++###^
Monounsaturated fat (En%)	5.58 ± 0.02 ^b^	6.32 ± 0.04 ^a^	5.01 ± 0.02 ^c^	5.97 ± 0.02 ^b***+++##^
Polyunsaturated fat (En%)	3.14 ± 0.02 ^b^	3.61 ± 0.04 ^a^	2.89 ± 0.02 ^b^	3.40 ± 0.02 ^b***+++^
Cholesterol (mg/day)	146 ± 0.82 ^c^	201 ± 1.57 ^a^	156 ± 0.69 ^b^	204 ± 0.81 ^a***+++##^
Fiber (g/day)	14.1 ± 0.07 ^b^	15.2 ± 0.13 ^a^	14.3 ± 0.06 ^b^	15.6 ± 0.07 ^a**+++^
Calcium (mg/day)	356 ± 1.56 ^d^	516 ± 2.9 7^b^	402 ± 1.32 ^c^	570 ± 1.54 ^a***+++^
Sodium (g/day)	2.43 ± 0.01 ^a^	2.47 ± 0.02 ^a^	2.38 ± 0.08 ^b^	2.46 ± 0.10^a *+++^
Vitamin C (mg/day)	83.9 ± 0.47 ^d^	117 ± 0.89 ^b^	97.5 ± 0.39 ^c^	136 ± 0.46 ^a***+++###^
Vitamin D (ug/day)	4.61 ± 0.04 ^d^	7.85 ± 0.08 ^b^	5.67 ± 0.03 ^c^	8.96 ± 0.04 ^a***+++^
DII (scores)	−17.5 ± 0.12 ^d^	−22.0 ± 0.23 ^b^	−18.7 ± 0.10 ^c^	−23.5 ± 0.12 ^a***+++^
Flavonoids (mg/day)	27.1 ± 0.24 ^d^	44.3 ± 0.46 ^b^	34.5 ± 0.20 ^c^	54.1 ± 0.24 ^a***+++###^
KBD (N, %)	6319 (39.3)	1782 (42.5) ^‡‡‡^	5657 (24.6)	5811 (37.8) ^‡‡‡^
PBD (N, %)	16,095 (41.1)	4198 (21.4)	23,030 (58.9)	15,378 (78.6) ^‡‡‡^
WSD (N, %)	8255 (51.3)	2176 (51.8)	7188 (31.2)	5933 (38.6) ^‡‡‡^
RMD (N, %)	5017 (31.2)	1448 (34.5) ^‡‡‡^	7079 (30.7)	6027 (39.2) ^‡‡‡^

The values represent the number of subjects (percentage of each group) for categorical variables and adjusted means ± standard deviations for continuous variables. Covariates included age, gender, residence area, income, education, body mass index, energy intake, physical activity, and smoking status. EER, estimated energy requirement; CHO, carbohydrates; DII, dietary inflammatory index; KBD, Korean-style balanced diet; PBD, plant-based diet; WSD, Western-style diet; RMD, rice-main diet. ^‡‡‡^ Significant difference from the high PBD group in a categorical variable for each gender at *p* < 0.001. * Significant differences from the male group at *p* < 0.05, ** *p* < 0.01, *** *p* < 0.001. ^+++^ Significant differences from the low PBD intake group at *p* < 0.001 ^#^ Significant interaction between gender and PBD intake group at *p* < 0.05, ^##^ at *p* < 0.01, ^###^ *p* < 0.001. ^a,b,c,d^ Means with different superscript letters were found to be significantly different by Tukey’s test, at *p* < 0.05.

**Table 3:** Metabolic parameters according to gender and a plant-based diet intake (PBD) status.

	Men		Women		Adjusted Odds Ratio and 95% CI
	Low PBD (*n* = 8841)	High PBD (*n* = 11,452)	Low PBD (*n* = 10,627)	High PBD (*n* = 27,781)	
MetS (Yes, %) ^1^	2884 (17.9)	714 (17.0)	3137 (13.6)	1565 (10.2) ^‡‡‡^	0.832(0.780–0.887)
BMI (mg/kg^2^) ^2^	24.4 ± 0.02 ^a^	24.3 ± 0.04 ^a^	23.7 ± 0.02 ^b^	23.4 ± 0.02 ^c***+++###^	0.836(0.796–0.877)
Waist C. (cm)^3^	85.5 ± 0.07 ^a^	84.9 ± 0.13 ^b^	78.6 ± 0.06 ^c^	77.6 ± 0.07^d ***+++#^	0.819(0.778–0.862)
SMI (kg/m^2^) ^4^	7.25 + 0.01 ^a^	7.24 + 0.01 ^a^	6.08 + 0.01 ^b^	6.02 + 0.01 ^c***+++##^	0.884(0.821–0.952)
Fat mass (%) ^5^	23.1 + 0.04 ^c^	22.9 + 0.06 ^d^	31.3 + 0.03 ^b^	30.9 + 0.03 ^b***+++##^	0.883(0.821–0.950)
Serum glucose (mg/dL) ^6^	98.6 ± 0.17 ^a^	97.7 ± 0.33 ^b^	93.7 ± 0.14 ^c^	93.0 ± 0.17 ^c**+###^	0.913(0.857–0.974)
HbA1c (%) ^7^	5.75 ± 0.01 ^a^	5.72 ± 0.02 ^a^	5.7 0± 0.01 ^b^	5.68 ± 0.01 ^b***+^	0.830(0.749–0.921)
Insulin resistance (%) ^8^	1860 (11.6)	452 (10.8)	1542 (6.70)	750 (4.88) ^‡‡‡^	0.776(0.697–0.864)
Serum total chol (mg/dL) ^9^	191 ± 0.31 ^b^	189 ± 0.58 ^c^	201 ± 0.26 ^a^	201 ± 0.31 ^a***+^	1.009(0.962–1.059)
Serum HDL (mg/dL) ^10^	49.3 ± 0.11 ^c^	49.5 ± 0.21 ^c^	55.6 ± 0.09 ^b^	56.9 ± 0.11 ^a***+++###^	1.173(1.121–1.226)
Serum LDL (mg/dL) ^11^	113 ± 0.29 ^b^	113 ± 0.54 ^b^	122 ± 0.24 ^a^	122 ± 0.28 ^a***^	1.024(0.969–1.082)
Serum TG (mg/dL) ^12^	146 ± 0.73 ^a^	133 ± 1.38 ^b^	117 ± 0.61 ^c^	112 ± 0.71 ^d***+++##^	0.871(0.832–0.913)
SBP (mmHg) ^13^	125 ± 0.12 ^a^	124 ± 0.23 ^a^	121 ± 0.10 ^b^	121 ± 0.11 ^b***+^	0.992(0.949–1.037)
DBP (mmHg) ^14^	77.8 ± 0.08 ^a^	77.5 ± 0.15 ^a^	74.7 ± 0.07 ^b^	74.6 ± 0.09 ^b***+^	0.974(0.906–1.047)
Serum C-reactive protein (mg/L) ^15^	0.158 ± 0.004 ^a^	0.164 ± 0.0074 ^a^	0.131 ± 0.003 ^b^	0.127 ± 0.0043 ^b***^	1.013(0.851–1.206)
eGFR (ml/min) ^16^	84.8 ± 0.13 ^c^	83.8 ± 0.25 ^d^	87.1 ± 0.11 ^a^	86.6 ± 0.13 ^b***+++^	1.081(1.018–1.148)
Serum AST (U/L) ^17^	25.1 ± 0.21 ^a^	24.7 ± 0.39 ^a^	23.1 ± 0.17 ^b^	23.0 ± 0.20 ^b***^	0.933(0.842–1.033) 0.993 (0.928 1.062)
Serum ALT (U/L) ^18^	26.0 ± 0.20 ^a^	25.3 ± 0.48 ^a^	20.5 ± 0.17 ^b^	20.5 ± 0.19 ^b***^	

Values represent mean ± standard errors (*n* = 10). ^*^ Significant differences by genders at *p* < 0.05, ^**^ at *p* < 0.01, ^***^ *p* < 0.001. ^+^ Significant differences by PBD intake at *p* < 0.05, ^++^ at *p* < 0.01, ^+++^ *p* < 0.001. ^#^ Significant interaction between genders and PBD intake, ^##^ at *p* < 0.01, ^###^ *p* < 0.001. ^a,b,c,d^ Means with different superscript letters in each variable represent significant differences among the groups, determined by the Tukey test, at *p* < 0.05. Adjusted odds ratio (OR) was calculated using logistic regression with low PBD intake as the reference. The cutoff points for the logistic regression were as follows. ^1^ Metabolic syndrome (MetS) criteria: ^2^ <25 kg/m^2^ for body mass index (BMI); ^3^ <90 cm for men and 85 cm for women for waist circumference (C.); ^4^ <7.4 kg/m^2^ for men and 6.2 kg/m^2^ for women for skeletal mass index (SMI), calculated by dividing skeletal muscle mass by height; ^5^ <25% for men and 30% for women for fat mass; ^6^ <126 mg/dL fasting serum glucose concentrations plus taking a hypoglycemic drug; ^7^ <6.5% hemoglobin A1c (HbA1c) plus diabetic drug intake; ^8^ <HOMA-IR for insulin resistance; ^9^ <230 mg/dL serum total cholesterol (chol) concentrations; ^10^ >40 mg/dL for men and 50 mg/dL for women for serum high density lipoprotein (HDL) cholesterol; ^11^ <160 mg/dL low-density lipoprotein (LDL) concentrations; ^12^ <150 mg/dL plasma triglyceride (TG) concentrations; ^13^ <140 mmHg systolic blood pressure (SBP), ^14^ <90 mmHg diastolic blood pressure (DBP) plus hypertension medication; ^15^ <0.5 mg/dL serum high-sensitive C-reactive protein (hs-CRP) concentrations; ^16^ estimated glomerular filtration rate (eGFR) <70 mL/min; ^17^ aspartate aminotransferase (AST) <40 U/L; ^18^ alanine aminotransferase (ALT) <35 U/L. CI, confidence intervals.

**Table 4:** Association of plant-based diet (PBD) intake with metabolic syndrome (MetS), including its traits, using two-sample Mendelian randomization (MR).

Exposures	Two-Sample MR			Heterogeneity ^2^		Pleiotropy ^2^		
	Method	OR (95% CI) ^1^	*p*–Value	Q	*p*–Value	Intercept	SE	*p*–Value
MetS	MR-Egger	1.715 (0.453–6.487)	0.432	1.752	1	−0.007	0.026	0.778
	WMD	1.363 (0.931–1.997)	0.112					
	IVW	1.422 (1.047–1.930)	0.024	1.833	1			
	WMO	1.328 (0.588–3.001)	0.700					
Hypertension	MR-Egger	0.992 (0.2886–3.408)	0.990	1.281	1	0.010	0.024	0.668
	WMD	1.234 (0.870–1.749)	0.239					
	IVW	1.292 (0.973–1.716)	0.077	1.468	1			
	WMO	1.167 (0.569–2.392)	0.675					
Exposure	Two-sample MR			Heterogeneity		Pleiotropy		
	Method	β (95% CI) ^1^	*p*-value	Q	*p*–Value	Intercept	SE	*p*–Value
Waist circumference (cm)	MR-Egger	0.222 (−1.383–1.827)	0.788	2.746	1	0.010	0.031	0.758
	WMD	0.444 (0.027–0.991)	0.048					
	IVW	0.469 (0.100–0.837)	0.013	2.842	1			
	WMO	0.430 (−0.484–1.344)	0.362					
Serum glucose concentration (mg/dL)	MR-Egger	0.320 (−1.601–2.240)	0.746	2.335	1	0.009	0.038	0.817
	WMD	0.453 (−0.109–1.016)	0.114					
	IVW	0.542 (0.099–0.985)	0.017	2.389	1			
	WMO	0.413 (−0.657–1.482)	0.454					
Serum triglyceride concentration (mg/dL)	MR-Egger	0.703 (−0.779–2.186)	0.194	3.315	1	−0.011	0.029	0.709
	WMD	0.326 (−0.102–0.753)	0.136					
	IVW	0.427 (0.085–0.768)	0.014	3.456	1			
	WMO	0.218 (−0.708–1.144)	0.647					
Serum HDL concentration (mg/dL)	MR-Egger	0.287 (−0.942–1.514)	0.650	3.515	1	0.002	0.024	0.926
	WMD	0.287 (−0.077–0.651)	0.122					
	IVW	0.343 (0.061–0.626)	0.017	3.523	1			
	WMO	0.169 (−0.507–0.946)	0.556					

^1^ Reference was the PBD (≥75th percentile) in logistic regression. ^2^ Heterogeneity and pleiotropy, determined by the MR-Egger method. WMD, weighted median; IVW, inverse-variance weighting; WMO, weighted mode; SE, standard error.

## Data Availability

The datasets analyzed are available in the Korean biobank (Osong, Korea). The secondary data generated from the analysis are available from the corresponding author upon reasonable request.

## Supplementary Materials

The following supporting information can be downloaded at: https://www.mdpi.com/article/10.3390/foods12030545/s1, Table S1: Genetic characteristics of instrumental variables.
