# Supplementary material for: A Causal and Inverse Relationship between Plant-Based Diet Intake and in a Two-Sample Mendelian Randomization Study
Source: Foods. 2023 Jan 26;12(3):545. doi: 10.3390/foods12030545 (PMC9914273; doi:10.3390/foods12030545)
Supplement: Supplementary file 1 [file foods-12-00545-s001.zip › foods-2175644-supplementary.pdf]

Supplementary Table S1. Genetic characteristics of instrumental variables

	SNP	Chromos	Gene name	Risk allele	Non-risk a	beta	se	p val	MAF	MetS	Glucose	Waist
						PBD						
1	rs426706	1	AGBL4	G	T	-0.03217	0.01819	4.62E-05	0.6633	0.8573	0.5944	0.05698
2	rs12730380	1	ENSG00000233290	A	G	-0.04196	0.02013	1.56E-06	0.7546	0.7592	0.9089	0.179
3	rs2594678	1	None	A	G	-0.03452	0.01744	5.16E-06	0.5821	0.881	0.3677	0.09899
4	rs7581376	2	RASGRP3	A	G	0.03623	0.01974	2.51E-05	0.2415	0.8945	0.244	0.9329
5	rs11122900	2	ENSG00000286481	G	A	0.031408	0.01709	2.23E-05	0.488	0.5771	0.2383	0.7144
6	rs41493149	2	LRP1B	A	G	0.033424	0.01742	9.21E-06	0.4072	0.1634	0.5759	0.9266
7	rs10207472	2	None	A	G	-0.06869	0.03754	2.51E-05	0.94378	0.106	0.7601	0.534
8	rs4663424	2	None	G	A	-0.0355	0.01801	5.54E-06	0.6495	0.6314	0.1435	0.2869
9	rs372517574	3	SUMF1	G	C	0.098298	0.05558	4.68E-05	0.02271	0.663	0.4167	0.4772
10	rs62237505	3	SGO1-AS1	A	G	-0.08603	0.04816	3.88E-05	0.96492	0.3255	0.494	0.6952
11	rs6809021	3	None	G	C	0.033424	0.01896	4.94E-05	0.2794	0.9169	0.01729	0.8876
12	rs6763709	3	None	A	G	0.033021	0.0176	1.62E-05	0.3822	0.4947	0.5374	0.8267
13	rs16864262	3	MBNL1	A	T	-0.03391	0.0184	2.23E-05	0.6831	0.4213	0.6086	0.7239
14	rs559235	3	None	C	T	-0.03082	0.01728	3.96E-05	0.5447	0.8815	0.5262	0.3189
15	rs77335151	4	NIPAL1	G	A	0.074816	0.0381	5.90E-06	0.05085	0.4475	0.6151	0.4465
16	rs112852183	4	None	C	G	-0.06068	0.03288	2.13E-05	0.92375	0.2512	0.8312	0.3534
17	rs4711416	6	None	T	G	0.039811	0.0225	4.65E-05	0.1736	0.1917	0.2687	0.1898
18	rs75521460	9	None	A	G	0.049606	0.02644	1.64E-05	0.1154	0.4534	0.4677	0.6691
19	rs1922159	10	PCDH15	C	T	-0.03175	0.01711	1.93E-05	0.5245	0.6909	0.473	0.3159
20	rs644205	10	None	G	A	-0.05849	0.0293	4.25E-06	0.90176	0.1961	0.01911	0.2211
21	rs808425	10	None	G	T	0.03623	0.02026	4.11E-05	0.2264	0.5157	0.1181	0.4566
22	rs7116119	11	SLC22A9	C	T	0.03583	0.01896	1.21E-05	0.2768	0.2615	0.1388	0.1392
23	rs112382545	11	ETS1	A	G	0.05038	0.02725	2.19E-05	0.1079	0.144	0.5212	0.4999
24	rs7135559	12	PTPRQ	C	A	-0.03245	0.01758	2.14E-05	0.6075	0.547	0.3606	0.471
25	rs9509286	13	IFT88	T	G	0.032216	0.01792	3.89E-05	0.337	0.05646	0.2966	0.7691
26	rs7323767	13	None	A	G	-0.0318	0.018	4.78E-05	0.6482	0.1084	0.4323	0.713

27	rs9556416	13	SOX21-AS1	A	G	-0.04769	0.02682	4.22E-05	0.8809	0.3253	0.27	0.02921
28	rs9805972	14	LINC02307	T	C	0.041787	0.02212	1.26E-05	0.1795	0.594	0.9479	0.8336
29	rs7156733	14	SAMD4A	G	A	-0.05889	0.03324	4.52E-05	0.92547	0.3506	0.229	0.3395
30	rs11844430	14	None	T	C	-0.03306	0.01722	9.90E-06	0.5393	0.319	0.05507	0.5313
31	rs77956082	15	None	A	G	0.049606	0.02575	8.89E-06	0.1219	0.08589	0.8881	0.9342
32	rs12930358	16	RBFOX1	C	G	0.040207	0.02259	3.89E-05	0.1675	0.723	0.2881	0.3517
33	rs7185137	16	None	C	T	0.032216	0.01714	1.56E-05	0.4778	0.25	0.6352	0.5967
34	rs55747549	16	MEAK7	G	C	-0.03654	0.01992	2.39E-05	0.7512	0.8244	0.7325	0.6558
35	rs2637364	17	ASIC2	T	C	0.031408	0.01756	4.22E-05	0.3803	0.4163	0.161	0.8295
36	rs12455899	18	None	T	C	-0.04225	0.02398	4.99E-05	0.847	0.555	0.3192	0.2597
37	rs28628729	18	None	A	G	0.03583	0.02021	4.74E-05	0.2287	0.2568	0.6516	0.3574
38	rs6117294	20	CASC20	T	G	-0.0332	0.01881	4.87E-05	0.7079	0.3384	0.5871	0.3857
39	rs6095646	20	None	A	G	0.039017	0.02118	2.12E-05	0.2014	0.9802	0.8958	0.4464
40	rs6013304	20	None	A	C	-0.03863	0.01994	8.14E-06	0.7477	0.4732	0.8153	0.5697
41	rs6128258	20	None	C	T	0.037426	0.02048	2.82E-05	0.2202	0.2956	0.3893	0.5655
42	rs743492	21	TSPEAR	A	G	0.03623	0.01834	5.18E-06	0.3139	0.583	0.2909	0.5037

P value

BP	TG	AST/ALT	HDL
0.3107	0.2779	0.05698	0.4193
0.4558	0.2831	0.179	0.7844
0.7711	0.4979	0.09899	0.9246
0.2955	0.1453	0.9329	0.828
0.7685	0.8827	0.7144	0.396
0.1963	0.1037	0.9266	0.5741
0.4547	0.4902	0.534	0.1008
0.5937	0.8964	0.2869	0.2785
0.9904	0.444	0.4772	0.8883
0.6372	0.9575	0.6952	0.6553
0.09206	0.6176	0.8876	0.5745
0.1264	0.328	0.8267	0.5163
0.3177	0.8432	0.7239	0.5871
0.194	0.5821	0.3189	0.4389
0.575	0.9024	0.4465	0.9592
0.4861	0.004604	0.3534	0.1759
0.9314	0.8605	0.1898	0.1189
0.6615	0.09482	0.6691	0.442
0.8507	0.9105	0.3159	0.1041
0.7782	0.01475	0.2211	0.3271
0.4117	0.446	0.4566	0.6905
0.7309	0.7781	0.1392	0.8404
0.7677	0.2615	0.4999	0.9518
0.4224	0.2524	0.471	0.4909
0.8582	0.5921	0.7691	0.4422
0.1188	0.1691	0.713	0.3582

0.4493	0.5356	0.02921	0.5617
0.9494	0.2459	0.8336	0.8206
0.6527	0.4446	0.3395	0.155
0.6898	0.7326	0.5313	0.007766
0.8372	0.04029	0.9342	0.08622
0.4534	0.7227	0.3517	0.0716
0.5966	0.5161	0.5967	0.9778
0.8817	0.1767	0.6558	0.7906
0.3539	0.2773	0.8295	0.1385
0.9995	0.2894	0.2597	0.01807
0.374	0.9942	0.3574	0.962
0.5989	0.8375	0.3857	0.05375
0.409	0.894	0.4464	0.2708
0.313	0.5267	0.5697	0.2551
0.7221	0.0384	0.5655	0.3077
0.6427	0.2004	0.5037	0.9612
